# Suggestive Evidence for an Antidepressant Effect of Metreleptin Treatment in Patients with Lipodystrophy

**DOI:** 10.1159/000526357

**Published:** 2022-08-29

**Authors:** Diana Branco Vieira, Jochen Antel, Triinu Peters, Konstanze Miehle, Michael Stumvoll, Johannes Hebebrand, Haiko Schlögl

**Affiliations:** ^a^Department of Child and Adolescent Psychiatry, Psychosomatics and Psychotherapy, University Hospital Essen, University of Duisburg-Essen, Essen, Germany; ^b^Department of Child and Adolescent Psychiatry, Hospital Dona Estefânia, Centro Hospitalar Universitário de Lisboa Central, Lisbon, Portugal; ^c^Division of Endocrinology, Department of Endocrinology, Nephrology, Rheumatology, University Hospital Leipzig, Leipzig, Germany; ^d^Helmholtz Institute for Metabolic, Obesity and Vascular Research (HI-MAG) of the Helmholtz Zentrum München at the University of Leipzig and University Hospital Leipzig, Leipzig, Germany

**Keywords:** Lipodystrophy, Metreleptin, Leptin, Depression, Hypoleptinemia, Anorexia nervosa

## Abstract

**Introduction:**

Lipodystrophy (LD) syndromes are rare heterogeneous disorders characterized by reduction or absence of subcutaneous fat, low or nondetectable leptin concentrations in blood and impaired hunger/satiety regulation. Metreleptin treatment reverses metabolic complications and improves eating behavior in LD. Because depression in anorexia nervosa (AN), which is also characterized by hypoleptinemia, improves substantially upon treatment with metreleptin, we hypothesized that metreleptin substitution may be associated with an antidepressant effect in patients with LD, too.

**Methods:**

In this ancillary study, 10 adult patients with LD were treated with metreleptin. To assess depressive symptoms, the self-rating questionnaire Beck's Depression Inventory (BDI) was filled in at preestablished time points prior (T1) and after initiation of metreleptin (T2: 1 week; T3: 4 weeks; T4: 12 weeks) dosing. The differences between time points were tested with nonparametric Friedman's analysis of variance. Sensitivity analyses were performed upon exclusion of the BDI items addressing appetite and weight changes.

**Results:**

According to their BDI scores, 4 patients had mild depression and 2 had moderate depression at baseline. Friedman's test revealed significant differences in BDI scores between the four time points. Post hoc analyses revealed that the difference between T1 and T3 was significant upon Bonferroni correction (*p* = 0.034, effect size *r* = 0.88). The sensitivity analyses upon exclusion of the appetite and weight change items again revealed a significant Friedman's test and significant Bonferroni corrected differences in the revised BDI scores between T1 versus T2 (*p* = 0.002, *r* = 0.99) and T1 versus T3 (*p* = 0.007, *r* = 0.79).

**Discussion/Conclusion:**

Our study for the first time revealed suggestive evidence for an antidepressant effect of metreleptin in patients with LD. Metreleptin caused a rapid drop in depression scores within 1 week of treatment. A reduction of the depression score was also observed in 2 of the 3 LD patients whose BDI scores were in the normal range before start of the treatment. The reduction in total scores of BDI was still apparent after 3 months (T4) of dosing. This observation matches findings obtained in clinical case studies of AN patients, in whom depression scores also dropped during the first week of metreleptin treatment. It needs to be noted that by the nature of this observational study without a placebo group, nonspecific treatment expectation affecting mood cannot fully be ruled out.

## Introduction

Lipodystrophy (LD) syndromes are a group of rare heterogeneous disorders classified as orphan diseases and characterized by a reduction or absence of subcutaneous fat [1] with a selective deficiency in functional adipose tissue in the absence of nutritional deprivation or a catabolic state [2, 3, 4]. LD causes a reduced energy storage capacity and a deficiency of adipokines, among them leptin [2, 3, 4, 5]. As a consequence, lipids are stored ectopically in nonadipose tissue (e.g., liver, muscle, pancreas), leading to hypertriglyceridemia and major complications such as hepatic steatosis and cirrhosis [4, 6, 7]. Patients also often develop insulin resistance and diabetes. Taken together, patients with LD experience a range of often severe morbidities, which often severely impair quality of life (QoL) [5].

Adipose tissue is a metabolically active endocrine organ, secreting adipokines, such as adiponectin and leptin, that mediate the adipocyte-brain cross talk [8]. Patients with LD commonly have hypoleptinemia [3, 6] as a result of a lack of or reduced adipose tissue [7, 9]. LDs are categorized as genetic or acquired and by the distribution of fat loss, which can be generalized (GLD) or partial (PLD) [2, 3]. Those with generalized LD have extremely low serum concentrations of leptin, whereas they can range from low to high for partial LDs [6].

Leptin is a polypeptide hormone assumed to play a critical role in regulating body weight, food intake, and energy metabolism [10, 11]; an alternative hypothesis states that it is the major trigger for the adaptation to human starvation [12]. Leptin crosses the blood-brain barrier and binds to the long form of the leptin receptor that is distributed in several brain regions. These include hypothalamic and thalamic regions, but the receptor was also found in other brain regions such as the hippocampus, substantia nigra pars compacta, cerebellum, and some cortical areas [11, 13, 14]. This brain distribution of leptin receptor suggests that leptin may be involved in other neural functions besides regulating feeding behavior and energy expenditure [11].

Recombinant human methionyl leptin (metreleptin) is approved by the Food and Drug Administration as replacement therapy to prevent and treat complications of leptin deficiency in patients with congenital or acquired GLD [15]. This drug has also been approved for the treatment of GLD, confirmed familial partial LD, or acquired partial LD (Barraquer-Simons syndrome) in the EU [16, 17] and Japan [18]. Metreleptin can reverse metabolic complications [2, 6, 19] and improves eating behavior through a reduction of increased hunger feelings occurring in the leptin-deficient state [20, 21].

Recent studies suggest that leptin may have antidepressant properties and an underlying role in the regulation of mood [11, 14, 22]. Animal models of stress-induced depression have shown induction of low concentrations of leptin in the respective behavioral models of depression [23, 24, 25]. Application of exogenous leptin in stress induced rodent models in turn led to antidepressant effects [23, 26, 27, 28]. In humans, the relationship between leptin concentrations in blood and depression is viewed rather controversially across studies [8, 11, 14, 29]; confounders, including sex and body mass index, warrant particular attention. Meta-analyses have not found evidence for differences in leptin concentrations between patients with major depressive disorder and healthy controls (HC), even if subgroup analyses implicated both high and low leptin concentrations in patients with major depressive disorder when compared to HC [8, 29]. In a transdiagnostic study including patients with anorexia nervosa (AN), hypothalamic amenorrhea, and obesity in addition to HC, clinician-rated depression scores were markedly higher in those persons with the lowest leptin concentrations [22]; to our knowledge, this is the only study to simultaneously include patients with disorders (hypothalamic amenorrhea in addition to AN) entailing low leptin concentrations.

Up to now, only one larger study analyzed retrospectively the impact of metreleptin treatment of patients with GLD on QoL [5] but without applying standardized psychiatric instruments for the assessment of depression. There is currently only one case report describing the beneficial effects of metreleptin treatment of a 20-year-old female with GLD on QoL, anxiety, and depression scores [30]. An increased prevalence of mood, anxiety, pain, and eating disorders has been observed in female patients with non-HIV LD [31].

In this study, we investigated changes in depression scores upon initiation of metreleptin treatment in 10 treatment-naïve patients with LD. We hypothesized that depression scores would decline during treatment.

## Materials and Methods

### Recruitment

This ancillary study made use of previously ascertained data: the adult patients with LD were treated with metreleptin at the University Hospital Leipzig between 2010 and 2014. Patients were referred from specialists in internal medicine and endocrinology from Germany and Austria to the treatment center in Leipzig, which at the time was the only center offering metreleptin treatment to adult patients in these two countries.

Inclusion criteria for leptin replacement were diagnosis of LD, insufficiently controlled diabetes (i.e., hemoglobin A1c ≥8.0%), and/or hypertriglyceridemia (i.e., fasting plasma triglycerides ≥200 mg/dL) despite adequate antihyperglycemic and lipid-lowering medication, respectively. Exclusion criteria included pregnancy or lactation, renal insufficiency with an estimated glomerular filtration rate <40 mL/min/1.73 m^2^, active malignant disease, primary hematologic abnormalities, infectious liver disease, HIV infection, and hypersensitivity to *Escherichia coli*-derived proteins. All patients who were able to perform study procedures were asked to participate in the scientific assessments. They gave written informed consent for every study procedure. Participation in the investigational study was not a prerequisite for receiving metreleptin treatment.

### Patients

All patients were metreleptin treatment-naïve. Nine women and one man were included in the study, and all had PLD. Mean age at start of metreleptin treatment was 42.1 years. Baseline characteristics of included patients are displayed in Table 1. A subgroup of the patient sample has been previously described [20, 21]. None of our LD patients was on medical treatment with antidepressants, neither at the beginning nor during the first 12 weeks of metreleptin treatment. One patient received one singular psychological intervention due to adjustment disorder between T1 and T2. No patient was on psychotherapy because of depression.

### Medication

The recombinant leptin analogue metreleptin was used for treatment. At the time of treatment, metreleptin was in clinical development, but not yet approved [15, 17]. Metreleptin was provided by Amylin (San Diego, CA, USA) and the subsequent license holders (Bristol-Myers Squibb, Munich, Germany, AstraZeneca, London, UK, and Aegerion Pharmaceuticals, Cambridge, MA, USA) and administered subcutaneously. Dosing was performed as recommended by the respective manufacturer to achieve physiological replacement. Recommendations changed slightly during the course of the study. Metreleptin was administered subcutaneously, and daily doses were between 2.5 and 7.8 mg. A detailed description of administered doses for patients 1–9 can be found in Schlogl et al. [20]. Doses for patients 10–12 were 5 mg/day once daily, independent of body weight.

### Experimental Design

**Fig. 1:**
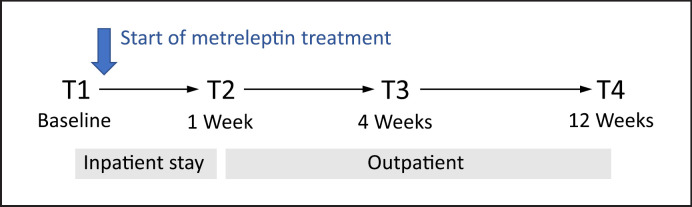
Time course of metreleptin treatment and study assessments.

The Beck's Depression Inventory (BDI) [32] was filled in at preestablished time points prior to and after initiation of metreleptin treatment to assess depressive symptoms. T1 was directly prior (1–2 days) to dosing day 1, and T2 after 1 week of initiation of metreleptin treatment. Between T1 and T2, patients were hospitalized; after T2, patients were discharged. T3 was about 4 weeks, T4 about 12 weeks, T5 about 26 weeks, and T6 about 52 weeks after the start of treatment. Since there were few patients overall in the study group and the later time points had more frequent missing data, we based the current dataset on those 10 adult patients with complete measurements at the four time points T1 to T4 (see Fig. 1). Anthropometric data such as height and body weight were collected at each time point.

### Psychological Measures

The BDI consists of 21 questions about how the person felt in the last week. Each question has a set of four possible answer choices, ranging in intensity [32]. A value of 0–3 is assigned for each answer; the total score is used to determine the depression severity. The standard cutoffs are as follows: 0–9, no or minimal depression; 10–18, mild to moderate depression; 19–29, moderate to severe depression; and 30–63, severe depression [33]. The German version of the BDI was used. In line with other rating scales for depression, the BDI does not measure a nosological entity. Instead, the BDI measures the intensity of depression by means of the main symptoms of the depressive syndrome [34].

We expected that treatment with metreleptin would reduce depressive symptoms independent of the effect on appetite and weight. Therefore, we performed sensitivity analyses, where questions R and S of the BDI were excluded to derive a revised total score. Question R refers to appetite and is scaled from “My appetite is no worse than usual” (0 points) to “I have no appetite at all” (3 points). Question S refers to weight loss and is scaled from “I have hardly lost any weight lately” (0 points) to “I have lost more than 8 kilos” (3 points). Maximum available score for this revised version of BDI was 6 points less than for the complete BDI.

### Biochemical Analysis

Blood was drawn in the fasted state at 8:00 a.m. After centrifugation, serum samples were then stored at −80°C until analysis. Leptin concentrations were assessed with a commercially available enzyme-linked immunosorbent assay (Mediagnost, Reutlingen, Germany), which was used according to the manufacturer's instructions.

### Data Analysis

Based on recent observations in patients with AN treated with metreleptin, we hypothesized that similar effects on depressive symptoms should be seen soon after treatment initiation in patients with LD. The differences between time points T1 to T4 were tested with nonparametric Friedman's analysis of variance (ANOVA) with post hoc analyses to be conducted in case of a significant result of the ANOVA. The *p* values of the post hoc analyses were corrected according to Bonferroni for six tests. Effect sizes for significant differences were calculated using the formula *r* = *z*/√*N*.

We calculated Spearman's correlations to test the relationship between leptin concentrations and BDI at T1. The leptin level was log-transformed.

Two-sided exact *p* values were calculated for all tests, the alpha level was set to 0.05. Analyses were carried out with IBM® SPSS® Statistics Version 27.0.0.0.

## Results

**Fig. 2:**
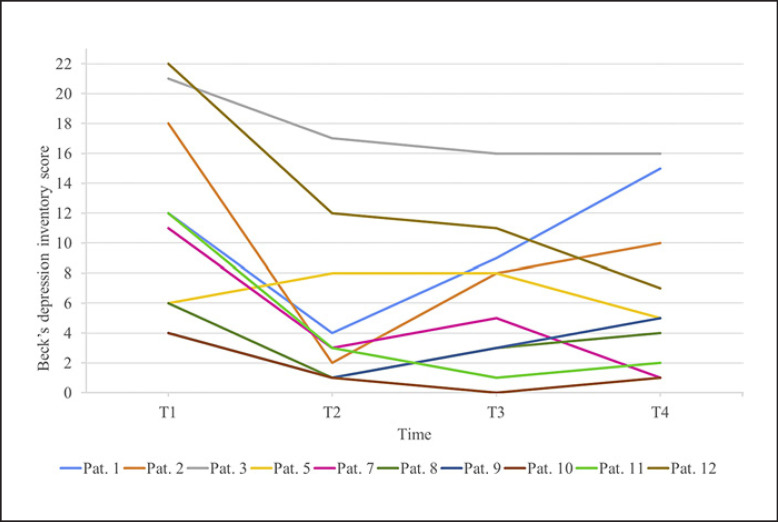
BDI scores of 10 patients with LD at T1 to T4 (single male patient: No. 7). Pat., patient; T1, baseline; T2, on average 8 days; T3, 31 days; and T4, 91 days after initiation of metreleptin treatment.

Descriptive data of the sample are presented in Table 2. BDI scores at baseline prior to initiation of treatment with metreleptin and at the subsequent three time points are shown in Table 3 and in Figure 2. Of 10 patients, four had mild depression (patient Nos. 1, 2, 7, and 11) and 2 had moderate depression (patient Nos. 3 and 12) at baseline (T1) according to the BDI cutoffs. Because the study was observational and had no placebo group, nonspecific treatment expectations affecting mood cannot fully be ruled out.

For analyses of time points T1 to T5 and T1 to T6, we were able to include 7 patients, 3 patients were lost to follow up. The complete data set is displayed in supplementary Figure S1 (for all online suppl. material, see www.karger.com/doi/10.1159/000526357).

**Fig. 3:**
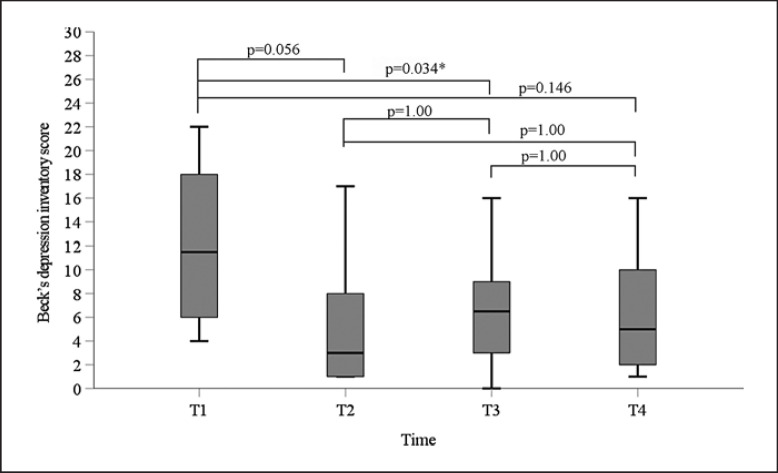
Boxplots for BDI scores of 10 patients with LD at baseline (T1) prior to initiation of metreleptin treatment and at T2–T4. Bonferroni-corrected *p* values of post hoc analyses (Friedman's test) are indicated (*p < 0.05). T1, baseline; T2, on average 8 days; T3, 31 days; and T4, 91 days after initiation of metreleptin treatment.

The nonparametric Friedman's test revealed significant differences in BDI scores between the four time points (χ^2^(df = 3) = 10.268; *p* = 0.012). Mean ranks were: T1 = 3.60, T2 = 2.10, T3 = 2.00, and T4 = 2.39. Post hoc analyses revealed that only the difference between T1 and T3 was significant upon Bonferroni correction (*p* = 0.034, effect size *r* = 0.88; shown in Fig. 3). Spearman's correlation coefficient between leptin serum concentrations (ln-transformed) and BDI at T1 was not significant (*ρ* = 0.04, *p* = 0.920; see online suppl. Fig. S2).

### Sensitivity Analyses

We conducted sensitivity analyses with BDI upon exclusion of scores for questions R and S. Again, the Friedman's test revealed significant differences in the revised BDI scores between time points T1 to T4 (χ^2^(3) = 12.41, *p* = 0.003). Post hoc analyses revealed significant differences in revised BDI scores between time points T1 versus T2 (Bonferroni corrected *p* = 0.002, *r* = 0.99) and T1 versus T3 (*p* = 0.007, *r* = 0.79).

## Discussion/Conclusion

Our study for the first time revealed an association of open-label metreleptin administration with decreased depressive symptoms in patients with LD, as deduced from the reduction of the BDI scores between baseline and the follow-ups during dosing. This substantiates our hypothesis that the antidepressant effect of leptin substitution in hypoleptinemia first observed in patients with AN extends to those with LD. Initial evidence suggests that patients with congenital leptin deficiency may also experience a mood improvement upon initiation of metreleptin treatment [35].

In the patients of the current study, total scores of BDI were significantly lower after initiation of metreleptin treatment, persisting up to T4 at 3 months after initiation of dosing. When considering the revised BDI scores, which excluded the items appetite and weight loss, the ANOVA was again significant and both the differences between T1 and T2 and T1 and T3, respectively, resulted in Bonferroni corrected *p* values of less than 0.05.

Like LD, AN is also characterized by hypoleptinemia. In AN, leptin acts as a key trigger for the body's adaptation to starvation by affecting diverse brain regions including the reward system [36]. Depressed mood is common in both starved persons [37] and patients with AN [38, 39] and tends to improve during refeeding [37, 40], which entails an increased leptin secretion [36]. Accordingly, we previously hypothesized that depression in patients with AN would improve upon treatment with metreleptin [36]. This hypothesis was supported by our recent case series of 3 female patients with AN who were treated with metreleptin, showing an antidepressant effect arising within 2 days of metreleptin treatment [41]. Another case report of a male adolescent with AN substantiated our hypothesis by revealing a substantial decrease in depressive symptoms after only 2 days of metreleptin treatment [42].

We were unable to identify a correlation between baseline leptin concentrations and corresponding BDI scores as previously reported for a larger sample encompassing patients with diagnoses of AN, hypothalamic amenorrhea or obesity, and HC [22]. The rapid drop in the depression score matches the findings obtained in clinical case studies of AN patients treated off-label with metreleptin [41, 42]. However, in contrast to AN patients, who all fulfilled diagnostic criteria for a comorbid diagnosis of a major depressive episode, in the current study a drop in the BDI score was also observed in 2 of the 3 LD patients whose BDI scores were in the normal range (<10 points; Fig. 2). Nine of the 10 patients had a lower BDI score at T2 in comparison to baseline (T1).

Our results only apply to the first 3 months of metreleptin treatment. It remains unclear, if the antidepressant effect persists over a longer time period (data shown in online suppl. Fig. S1). In a previous single case report, symptoms of depression in a patient with GLD were examined, namely prior to and after 3 and 6 months of initiation of metreleptin treatment [30]. The authors found an improvement in BDI scores at the third month of treatment with a slight worsening of this score at the sixth month of treatment, but overall improvement in symptoms compared to baseline. They also observed considerable improvements in different components of QoL.

In a study comprising 22 patients with PLD, mood disorders requiring medication were identified in 12 patients (52.2%). Depression was the most common mental disorder and occurred in 10 patients (43.5%) [43]. In a more recent study including 16 women with both GLD and PLD, a standardized diagnostic assessment was performed by two psychiatrists and the prevalence of mood disorders was 56% [31]. The authors concluded that patients with LD have an increased prevalence of mood, anxiety, pain, and eating disorders, which exceeded that of co-assessed patients with obesity or cancer. Patients with LD thus require a careful psychiatric evaluation and related long-term support [31]. The fact that 7 of the 10 patients of our study had a BDI score ≥10 points supports the finding of an elevated rate of mood disorders in LD patients [31, 43]. The rapid reduction of depressive symptoms induced by metreleptin suggests a causal role of hypoleptinemia for the development of depression in such patients.

Our previous findings suggestive of an antidepressant effect of metreleptin in patients with AN [41, 42] and data of this study in patients with LD are congruent with data of rodent models. In animals, systemic and intracerebroventricular injections of leptin produced antidepressant-like behavioral effects in stress-induced depression [23, 26, 27, 28], which has been associated with low leptin concentrations [23, 24, 25]. For humans, we hypothesize that the antidepressant effect of metreleptin applies to patients with hypoleptinemia.

Patients with LD have a reduced QoL presumably partially based on the burden of their disease, which apart from the metabolic complications may encompass the development of mental disorders as well as cosmetic issues related to ectopic fat distribution and as a consequence a negative body image [30, 44]. A recent study described retrospectively the impact of metreleptin on QoL [5]. The investigators used chart data, which were abstracted from a cohort of patients with GLD and PLD (*n* = 112) treated at the US National Institutes of Health in order to evaluate the effects of metreleptin treatment on patient outcomes and QoL [5]. They concluded that metreleptin treatment reduced the gap in QoL between untreated GLD and untreated PLD versus perfect health by approximately 59% and 31%, respectively [5]. Our data suggest that metreleptin treatment may reduce depressive symptoms, which might also contribute to an improved QoL rating.

Strengths of this study include being the first study in treatment-naïve patients with LD to examine the effects of metreleptin on depression scores over a 3-month period. To our knowledge, no study with *n* > 1 has been published so far in metreleptin treatment-naïve patients with LD analyzing depression measures. The pretreatment depression scores obtained with BDI were in the moderate to severe range in only 2 of the patients. In light of the inclusion of only 10 patients, it was all the more surprising that a significant reduction in depression scores was detectable; patients with no or a mild depression contributed to the antidepressant effect.

However, several limitations warrant consideration. First, in our study we only assessed BDI as a marker for depression. Further studies are needed to investigate if other psychopathologies observed in GLD or PLD [31, 43] also improve upon treatment with metreleptin. Second, the antidepressant effect of metreleptin in LD could be a primary effect on, for example, the dopaminergic system or secondary, for example, due to reversal of metabolic complications, improved eating behavior and less preoccupation with food, or both. Further studies are needed to clarify the exact mechanisms. Also, because the study lacked a placebo group, nonspecific treatment expectation affecting depressive symptoms cannot fully be ruled out, and the potential for a Hawthorne effect on subjective measures like the BDI is large. However, a potential treatment effect on depressive symptoms was not part of the information provided to the patients. Finally, the data were analyzed retrospectively to test a hypothesis based on findings obtained in patients with AN; the study was not designed to specifically detect an antidepressant effect of metreleptin.

In summary, the initiation of metreleptin treatment in patients with LD rapidly reduced self-reported depressive symptoms. These observations match well with recent findings about improvement of QoL through metreleptin in LD patients [5] and BDI scores and QoL in a case report of a GLD patient [30]. These effects of metreleptin treatment on depressive symptoms in LD patients fit with results reported for patients with AN [41, 42]. Taken together with data from patients with AN and a case report of a patient with congenital leptin deficiency [35], our results suggest that metreleptin may have a strong antidepressant effect in patients with hypoleptinemia, irrespective of the cause of leptin deficiency.

## Statement of Ethics

The Ethics Committee of the University of Leipzig approved this research project (approval No. 147/10-ek), according to the national research ethics regulations. All participants gave their written consent for all study procedures.

## Conflict of Interest Statement

Johannes Hebebrand and Jochen Antel declare that they are named as inventors in a patent application that the University of Duisburg-Essen filed on the use of leptin analogues for the treatment of depression; Johannes Hebebrand and Jochen Antel were also named as inventors in a patent application filed by the UDE for the use of leptin analogues for the treatment of anorexia nervosa; and Johannes Hebebrand received a speaker's honorarium from Amryt Pharmaceuticals in 2021. Konstanze Miehle and Haiko Schlögl declare that they have consulted for Aegerion Pharmaceuticals. All other authors declare that the research was conducted in the absence of any commercial or financial relationships that could be construed as a potential conflict of interest.

## Funding Sources

We thank the patients for their participation in the study. This work was supported by the Federal Ministry of Education and Research (BMBF), Germany, FKZ: 01EO1501 (IFB Adiposity Diseases); by the Deutsche Forschungsgemeinschaft (DFG; SFB 1052/2, A01 to MS); and by the Deutsches Zentrum für Diabetesforschung (DZD; Grant No. 82DZD00601).

## Author Contributions

Diana Vieira, Jochen Antel, Peter Tri-inu, and Johannes Hebebrand were involved in the study design, data analysis and interpretation, and writing and reviewing the final draft; Konstanze Miehle and Michael Stumvoll were involved in the study design and data collection and reviewing the final draft; and Haiko Schlögl was involved in the study design, data collection, and writing and reviewing the final draft. All of the authors participated in developing and reviewing the manuscript. All authors approved the final manuscript.

## Data Availability Statement

The data that support the findings of this study are not publicly available due to data protection rules. For further information, please contact HS (haiko.schoegl@medizin.uni-leipzig.de).

## Supplementary Material

Supplementary data

## Figures and Tables

**Table 1:** Baseline characteristics of LD patients included in the study

Patient	Mutation	Sex	Age, years	BMI, kg/m^2^	Leptin, ng/mL	HbA1c, mmol/mol (%)	TG, mmol/L
1	LMNA	F	23	27.4	5.3	6.4 (46)	21.1
2	LMNA	F	34	28.7	5.2	8.9 (74)	2.4
3	LMNA	F	48	27.4	9.8	6.6 (49)	2.5
5	ND	F	41	30.0	3.6	8.0 (64)	7.9
7	PPARy	M	55	33.5	6.7	6.0 (42)	23.2
8	ND	F	39	30.8	11.2	7.6 (60)	15.6
9	PPARy	F	33	27.2	5.6	7.0 (53)	4.3
10	LMNA	F	52	28.3	3.4	8.6 (70)	1.5
11	LMNA	F	45	25.0	2.3	9.2 (77)	2.8
12	LMNA	F	26	33.9	3.9	7.3 (57)	41.5

All patients phenotypically had partial LD; ethnicity was for all patients European/white. BMI, body mass index; F, female; HbA1c, glycated hemoglobin A1c; LD, lipodystrophy; LMNA, lamin-A/C; M, male; ND, not detected; PPARy, peroxisome-proliferator-activated receptor γ; TG, triglycerides.

**Table 2:** Descriptive data for 10 patients (nine women) with LD at baseline prior to initiation of metreleptin treatment (T1) and at time points T2-T4

		Mean (SD), range
Age, years	T1	42.1 (11.2), 23.0–55.3
Days between	T1 and T2	7.7 (2.8), 6–15
	T1 and T3	31.1 (5.4), 27–42
	T1 and T4	91.1 (6.3), 83–99
BMI, kg/m^2^	T1	29.2 (2.9), 25–33.9
	T2	28.8 (2.8), 24.2–33.3
	T3	29.0 (2.9), 24.8–33.8
	T4	29.0 (2.9), 24.2–33.9

BMI, body mass index; SD, standard deviation.

**Table 3:** BDI scores for 10 patients with LD at T1 to T4

Time point	BDI score			
	mean (SD), range	25th percentile	50th percentile	75th percentile
T1	11.6 (6.8), 4–22	5.5	11.5	18.8
T2	5.2 (2.5), 1–17	1.0	3.0	9.0
T3	6.4 (4.9), 0–16	2.5	6.5	9.5
T4	6.6 (6.4), 1–16	1.8	5.0	11.3

SD, standard deviation; T, time point; T1, baseline; T2, on average 8 days; T3, 31 days; and T4, 91 days after initiation of metreleptin treatment.

## Notes

Johannes Hebebrand and Haiko Schlögl: shared senior authorship.
