# Supplementary material for: Suggestive Evidence for an Antidepressant Effect of Metreleptin Treatment in Patients with Lipodystrophy
Source: Obes Facts. 2022 Aug 29;15(5):685–93. doi: 10.1159/000526357 (PMC9669995; doi:10.1159/000526357)
Supplement: Supplementary file 1 — Supplementary data [file ofa-0015-0685-s01.docx]

**Supplementary Data**

**Suggestive evidence for an antidepressant effect of metreleptin treatment in patients with lipodystrophy**

**
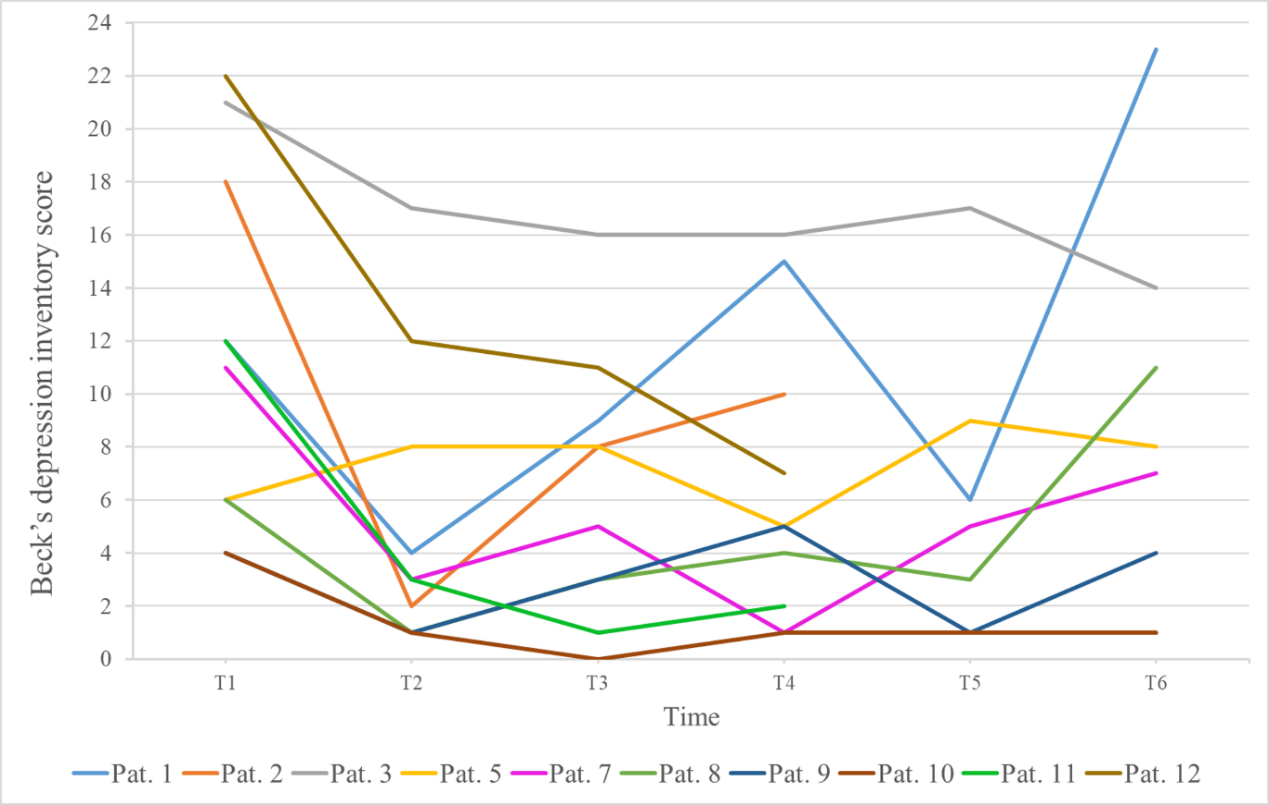
**

**Fig. S1.** Beck’s Depression Inventory scores for patients with lipodystrophy from T1 to T4 (n=10) and from T5 to T6 (n=7). T1, baseline; T2, on average 8 days; T3, 31 days; T4, 91 days, T5, 181 days; and T6, 360 days after initiation of metreleptin treatment.


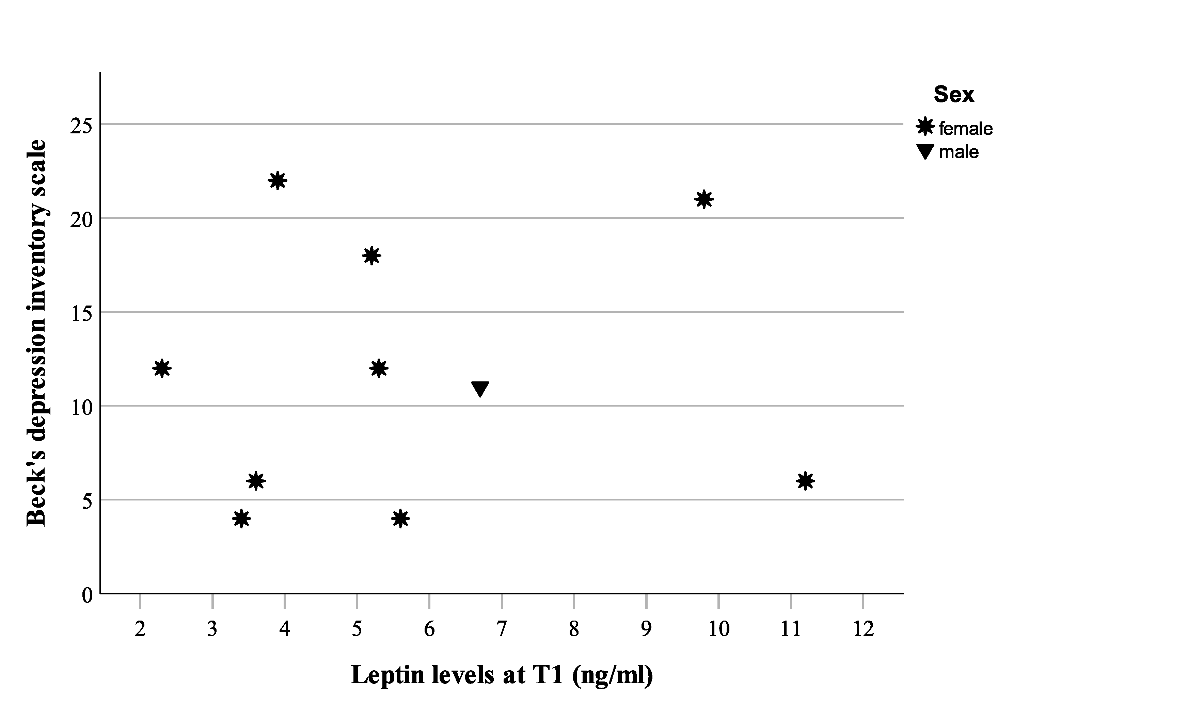


**Fig. S2**. Leptin serum concentrations and Beck’s Depression Inventory scores at T1 for patients (n=10) with lipodystrophy.
